# High-density SNP-based QTL mapping and candidate gene screening for yield-related blade length and width in *Saccharina japonica* (Laminariales, Phaeophyta)

**DOI:** 10.1038/s41598-018-32015-y

**Published:** 2018-09-11

**Authors:** Xiuliang Wang, Zhihang Chen, Qiuying Li, Jie Zhang, Shun Liu, Delin Duan

**Affiliations:** 1 Key Lab of Experimental Marine Biology, Institute of Oceanology, Chinese Academy of Sciences, Qingdao, 266071 China; 2 Lab for Marine Biology and Biotechnology, Qingdao National Laboratory for Marine Science and Technology, Qingdao, 266071 China; 3 University of Chinese Academy of Sciences, Beijing, 100093 China; 4 Center for Ocean Mega-Science, Chinese Academy of Sciences, 7 Nanhai Road, Qingdao, 266071 China

## Abstract

*Saccharina japonica* is one of the most important marine crops in China, Japan and Korea. Candidate genes associated with blade length and blade width have not yet been reported. Here, based on SLAF-seq, the 7627 resulting SNP loci were selected for genetic linkage mapping to 31 linkage groups with an average spacing of 0.69 cM, and QTL analyses were performed to map the blade length and blade width phenotypes of *S*. *japonica*. In total, 12 QTLs contributing to blade length and 10 to width were detected. Some QTL intervals were detected for both blade length and width. Additive alleles for increasing blade length and width in *S*. *japonica* came from both parents. After the QTL interval regions were comparatively mapped to the current reference genome of *S*. *japonica* (MEHQ00000000), 14 *Tic*20 (translocon on the inner envelope membrane of chloroplast) genes and three peptidase genes were identified. RT-qPCR analysis showed that the transcription levels of four *Tic*20 genes were different not only in the two parent sporophytes but also at different cultivation times within one parent. The SNP markers closely associated with blade length and width could be used to improve the selection efficiency of *S*. *japonica* breeding.

## Introduction

*S*. *japonica* is brown seaweed originally distributed along the coast of the Japan Sea and the Okhotsk Sea that is cultivated on a large scale^1–3^. It is typical giant kelp with a life history alternating between macro-sporophytes and micro-gametophytes^4^. Gametophytes can be easily isolated and preserved in the laboratory and used to produce new hybrids. The sporophyte of *S*. *japonica* is composed of a blade (frond), stipe and holdfast. After one year of cultivation in China, blade length is usually 2~4 meters, and the width is approximately 20~50 cm. This kelp is used mainly as human food, marine animal feed, and raw material for extracting alginate, mannitol and iodine^5,6^.

A total of 5.6 million tons (wet weight) of *S*. *japonica* was produced worldwide in 2012, mainly in China^7^. Although cultivation areas can be enlarged to some extent, kelp production in China relies partly on the application of newly developed varieties to increase productivity^7^. In breeding, the kelp blade length and width are always targeted for selection. Since the 1950s, many new kelp varieties with longer or wider blades have been obtained, such as “Haiqing No. 2”^8^, “860”^9^, “901”^10^. “Huangguan No. 1”^11^, “Dongfang No. 7” and “Rongfu”^12,13^. Nevertheless, the breeding process is often costly and labor intensive, and the development of one new kelp variety generally takes 5~6 years or more^14^. For efficient breeding, quantitative inheritance of blade length, blade width and stipe length has been studied in *S*. *japonica*^15–17^. However, little is known about the individual genes and their networks in relation to most quantitative traits, especially yield-related blade length and width.

Recently, attempts have been made to use molecular markers to construct genetic linkage map and perform quantitative trait locus (QTL) mapping for *S*. *japonica*^18–22^. In the two QTL mapping studies of *S*. *japonica* to date, several QTLs have been mapped for kelp blade length and width, raw weight and base shape^20,22^. The linkage maps were mainly constructed with moderate-throughput DNA markers, such as AFLPs and SSRs, with average genetic distances ranging from 6.7 cM to 7.92 cM. To improve QTL mapping efficiency for *S*. *japonica*, high-density genetic linkage maps are desirable, and these are best constructed using SNP markers.

SNP markers are di-allelic and represent the smallest type of genetic variation in genomes; they have been extensively applied for genetic diversity detection, genetic linkage map construction, QTL mapping, and GWAS^23^. Due to the comparatively high cost of resequencing, several methods such as RAD-seq (restriction associated DNA sequencing), 2b-RAD, and GBS (genotyping by sequencing), have recently been developed to discover additional SNPs and genotype large sample sets for non-model species^24^. SLAF-seq (specific-locus amplified fragment sequencing) is another such method and has a better balance of cost-efficiency and SNP detection than do other methods^25^. Recently, SLAF has been used to construct genetic linkage maps for other economically seaweeds, such as *Pyropia haitanensis*^26^, *Undaria pinnatifida*^27^. Zhang *et al*.^28^ constructed a genetic linkage map for *S*. *japonica* with an average genetic distance of 0.36 cM based on SNP markers. However, it was used to map a sex determination locus in *S*. *japonica* and not to map QTL for blade length and width^28^.

In this study, we conducted QTL mapping for *S*. *japonica*’s blade length and width based on a high-density genetic linkage map constructed with SNP markers that we developed using SLAF-seq. Based on a new *S*. *japonica* draft genome sequence (MEHQ00000000), fourteen *Tic*20 genes and three peptidase genes were found to be potential candidate genes associated with blade length and width in *S*. *japonica*. Our aim is to combine the collinearity of the high-density genetic linkage map with the reference genome to identify candidate genes for the yield-related blade length and width traits of *S*. *japonica*.

## Materials and Methods

### Construction of BC_1_F_2_ mapping population

The kelp germplasm used in this study was maintained at the Key Laboratory of Experimental Marine Biology of Institute of Oceanology, Chinese Academy of Sciences, Qingdao^29^. The “860” variety of *S*. *japonica* and the species *S*. *longissima* were selected as parents for construction of the mapping population. Variety “860” has ever been used extensively for cultivation and has a blade length of 240.2 ± 23.1 cm and a blade width 25.4 ± 3.0 cm^10^. *S*. *longissima* has a longer blade of usually 7~8 meters; thus, the species has often been used to breed new hybrids^11,30^. In brief, the male gametophyte clone of “860” was crossed with the female gametophyte clone of *S*. *longissima* to produce the F_1_ hybrid generation. Sporelings were cultured in the laboratory until the blade length reached 1~2 cm, after which the seedlings were moved to sea cultivation. When the F_1_ hybrids reached maturity in August, female and male gametophyte clones were isolated from one F_1_ hybrid sporophyte and preserved. Then, the “860” male gametophyte was crossed with F_1_ hybrid female gametophyte clones to develop the BC_1_F_1_ population. One mature sporophyte was chosen from the BC_1_F_1_ population and then self-fertilized in the laboratory to produce the BC_1_F_2_ mapping population, which was cultivated in the sea (E 122°62′, N 37°22′). During the cultivation, the blade length and width of 178 individuals were measured on April 17th, May 8th, May 22th and June 9th. The blade length was measured from the top of the stipe to the blade tip. The width was measured at the widest part of the blade.

### DNA extraction

The gametophytes from the male parent, “860”, and the female parent, *S*. *longissima*, as well as the 178 sporophytes from the BC_1_F_2_ mapping population were used for genomic DNA isolation. Each sample was ground into a powder using a high-speed homogenizer, and genomic DNA was extracted and purified according to the improved CTAB (cetyltrimethyl ammonium bromide) method^31^. The purity and concentration of the extracted DNA were determined using both an ND-1000 spectrophotometer (NanoDrop, Wilmington, DE, USA), and electrophoresis in an 0.8% agarose gel with a lambda DNA standard marker.

### SLAF library preparation and sequencing

The parental gametophytes and 178 sporophyte progeny were genotyped using the reported SLAF method with minor modifications^25^. The draft genome sequences of the kelp were in silico digested (GenBank Accession Number: MEHQ00000000). *Hae*III and *Rsa*I were selected as the fittest restriction endonucleases for SLAF analysis. For SLAF library construction, the extracted genomic DNA was first incubated at 37 °C with *Hae*III (New England Biolabs, NEB), T4 DNA ligase (NEB), and *Hae*III adapter. Then, *Rsa*I was added for additional digestion at 37 °C. The reaction-ligation reactions were inactivated by incubating at 65 °C. Then, PCR was performed using diluted restriction-ligation samples, dNTPs, *Taq* DNA polymerase (NEB) and PCR primers containing barcode 1. The PCR product were purified with Agencourt AMPure XP beads (Beckman Coulter, High Wycombe, UK) and pooled. The pooled sample was purified with a Quick Spin column (Qiagen) and then separated by electrophoresis on a 2% agarose gel. Fragments with sizes of 314~414 bp were excised and purified using a gel extraction kit (Qiagen, Hilden, Germany). Then, the purified products were subjected to PCR amplification with Phusion Masters Mix (NEB) and Solexa Amplification primers mix to add barcode 2. Finally, paired-end sequencing of 2 × 100 bp was performed on the selected SLAFs using an Illumina high-throughput sequencing platform (Illumina, Inc., USA). After sequencing, the Q30 and nucleotide compositions of the raw data were checked for sequencing quality.

### Development of SNP markers from the SLAF reads and genotyping

The separate fastq files of SLAF reads for each kelp DNA sample were aligned to the draft reference genome of *S*. *japonica* (MEHQ00000000) via the program BWA^32^, with a maximum of 4% sequence mismatch allowed. The alignments were transformed to the SAM (sequence alignment map) format, combined and assigned into one master BAM (binary file of SAM) format using SAMtools 0.1.18^33^. SNPs were detected with the genome analysis toolkit (GATK) using the BAM file; detailed manual for GATK analysis can be found at the website: https://software.broadinstitute.org/gatk/guide/.

The alleles belonging to each SNP marker were evaluated to determine minor allele frequency. For mapping populations of diploid sporophytes of kelp, one SNP locus can contain no more than four genotypes; thus, loci with more than four putative alleles were considered likely to originate from repetitive genome regions and excluded from the subsequent analysis. Therefore, only those SNP loci with 2~4 SLAFs were considered potential markers for construction of linkage maps in kelp. These polymorphic SNP markers were coded into eight segregation patterns as aa × bb, ab × cd, ef × eg, hk × hk, lm × ll, nn × np, ab × cc and cc × ab. Here, the segregation pattern aa × bb was used for genetic linkage map construction.

### Construction of linkage map and detection of QTLs for blade length and width

Before construction of the linkage map, the SNP markers were filtered for quality, requiring genotype calls at each SNP to have a depth of coverage of at least four reads in each individual kelp sporophyte and simultaneously excluding markers of significant segregation distortion (P < 0.05). Moreover, the segregation distortion for the SNP markers used for construction of linkage map was also examined with the software DistortedMap^34^.

HighMap was used for genetic linkage map construction. Pairwise modified logarithm of odds (MLOD) scores were used to partition mapping markers into LGs (linkage groups), and those markers with MLOD values < 5 were excluded from marker ordering. For the iterative process of marker ordering, a combination of enhanced Gibbs sampling, spatial sampling and simulated annealing algorithms was applied. The SMOOTH strategy was adopted for error correction according to the parental genotypes. The Kosambi mapping function was used to calculate the map distances between markers. Moreover, a heat map and a haplotype map were constructed to assess the quality of the genetic map^35,36^.

QTLs underlying blade length and blade width were mapped using QTL IciMapping 4.1.0.0 (http://www.isbreeding.net) with the inclusive composite interval mapping module^37^. QTLs with additive and dominance effects were examined with the method ICIM-ADD. The parameters for ICIM-ADD were set as a 1.0 cM scan step, a 0.001 probability used for stepwise regression analysis, 2.5 used for the LOD threshold score for significant QTLs for each trait. Furthermore, QTLs underlying blade length and blade width were also detected with the software QTL.gCIMapping from the R website^38^.

### Comparative mapping of candidate genes in detected QTLs

SLAF reads corresponding to the SNP markers in detected QTLs were used in a BLAST analysis against the kelp draft genome sequence (MEHQ00000000). The candidate genes were annotated and analyzed via the NCBI database (http://www.ncbi.nlm.nih.gov).

### RNA extraction and RT-PCR analysis

Blades of the parental sporophytes were collected on the four observation days for RNA extraction. Total RNA was extracted following the method of Yao *et al*.^39^. The extracted RNA was treated with RNase-free DNase I (TaKaRa, Dalian, China) to remove residual genomic DNA. First-strand cDNA was prepared with the PrimeScript II cDNA synthesis kit (Takara, Tokyo, Japan) according to the manufacturer’s instructions and then stored at −80 °C.

Transcriptional analysis of the four *Tic*20 genes from the parental sporophytes was conducted with real-time quantitative PCR (RT-qPCR). Primer pairs specific to the UTRs (untranslated region) of each of the four *Tic*20 genes, were used to amplify one 150 -bp amplicon per gene (Table S1). The β-actin primers qActin-F (5′-GACGGGTAAGGAAGAACGG-3′) and qActin-R (5′-GGGACAACCAAAACAAgggCAggAT-3′) were designed as internal controls.

RT-qPCR was performed with the SYBR Premix Ex *Taq* II kit (Takara, Tokyo, Japan) on the TP800 Thermal Cycler Dice (Takara, Tokyo, Japan). Thermal cycling protocol and data analysis were as previously reported^40^.

## Results

### SLAF analysis and SNP marker development and genotyping

In total, 364.11 M paired-end reads with a length of 200 bp were obtained from the 51.1 Gb of raw data from the SLAF library. The Q30 ratio was 87.04%, and the guanine/-cytosine content was 46.48%. In total, 13,567,809 reads were generated from the male parent (1.9 Gb of data) and 7,318,321 reads from the female parent (1 Gb). The average read number of the 178 individual sporophytes was 1,831,165. After the reads were mapped to the draft genome of kelp using BWA, we found that approximately 67.73% of total reads from the male parent, 44.18% from the female parent and 79.48% on average from each individual in the mapping population could be mapped to specific regions of the presumed chromosome regions. Ultimately, 1,365,570 polymorphic SLAFs were developed for the parents and the mapping population, of which 443,249 SLAFs were from the male parent, 226,614 from the female parent, and an average of 226,366 from the 178 BC_1_F_2_ individuals ranging from 117,848 to 587,670. Therefore, the average coverage for each SLAF marker was 30.6-fold in the male parent, 32.3-fold in the female parent, and an average of 8.0-fold in each individual of the mapping population.

Using SAMtools and GATK for realignment and detection, we identified a total of 1, 092, 944 SNPs from the 364.11 M original reads in the parent gametophytes and 178 BC_1_F_2_ sporophytes, of which 507, 994 SNPs appeared in the male parent, 330, 843 SNPs in the female parent, and an average of 292,415 in each of the 178 individuals of the mapping population.

After the low-depth SNPs were filtered out and the parental lines assigned different letters of alphabet to indicate their genotypes, we assessed SNP segregation patterns and found that 97, 189 SNPs out of 1, 092, 944 total polymorphic SNPs were successfully encoded and grouped into four segregation patterns (aa × bb, nn × np, lm × ll, hk × hk). Since the two parents were haploid gametophytes with genotypes of a and b, we selected 75,024 markers that followed the aa × bb segregation pattern for the subsequent linkage analysis.

### Genetic linkage map construction

**Figure 1:**
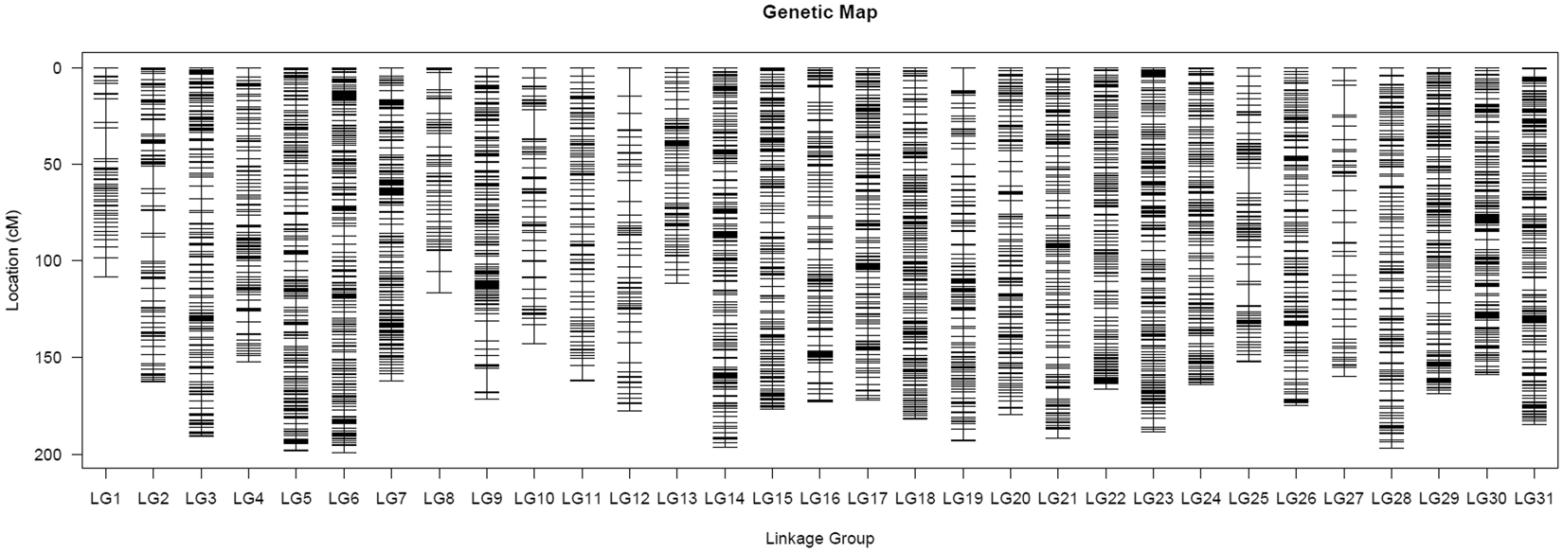
Genetic lengths and marker distribution of 31 linkage groups in genetic map of the kelp.

After incomplete markers and those showing significant segregation distortion markers were removed from the selected 75, 024 SNP markers, 7627 SNPs were finally retained for genetic map construction. All 7627 markers were assigned to the 31 LGs of kelp using HighMap (Fig. 1). The results from the software DistortedMap also showed that most of SNP markers in the 31 LGs have no segregation distortion, and the details of DistortedMap analysis for LGs 10, 11, 14 and 15 were listed in supplementary Excel file. The details of the SNP based genetic linkage map are listed in Table 1. The total genetic length of the SNP map was 5232.42 cM. The genetic distances of the 31 LGs spanned 108.51 cM (LG1)~199.40 cM (LG6), with mean marker intervals ranging from 0.42 cM (LG23) to 2.53 cM (LG27). The average distance between adjacent markers was 0.69 cM. The largest LG (LG6) contained 374 SNPs and was 199.4 cM long, while the smallest LG1 had 59 SNPs and was 108.51 cM long. Each LG had an average of 246 SNP markers and was 168.79 cM long. The maximum gap in each LG ranged from 4.09 cM to 15.96 cM; In most cases, the maximum gap was between 4 cM and 7 cM, although six LGs had maximum gaps of over 10 cM.

**Table 1:** Summary statistics of the genetic map of *S*. *japonica*.

Linkage group ID	Total marker	Total distance (cM)	Average distance (cM)	Max gap (cM)
LG1	59	108.51	1.84	15.96
LG2	146	162.32	1.11	12.58
LG3	242	190.75	0.79	6.27
LG4	158	152.36	0.96	6.03
LG5	397	198.09	0.50	5.09
LG6	374	199.40	0.53	5.02
LG7	322	162.21	0.50	4.62
LG8	66	116.37	1.76	11.02
LG9	239	171.35	0.72	12.44
LG10	76	142.56	1.88	14.9
LG11	134	161.86	1.21	7.39
LG12	80	177.69	2.22	14.62
LG13	113	111.69	0.99	5.07
LG14	392	196.30	0.50	4.56
LG15	308	176.50	0.57	5.42
LG16	219	172.96	0.79	8.17
LG17	315	172.07	0.55	4.28
LG18	377	181.80	0.48	4.09
LG19	226	192.90	0.85	11.98
LG20	224	179.28	0.80	7.81
LG21	233	191.76	0.82	5.7
LG22	388	166.10	0.43	4.56
LG23	445	188.21	0.42	4.9
LG24	357	164.06	0.46	4.23
LG25	142	152.03	1.07	13.1
LG26	201	174.82	0.87	4.53
LG27	63	159.58	2.53	15.8
LG28	239	196.96	0.82	5.61
LG29	329	168.67	0.51	6.72
LG30	358	158.55	0.44	5.19
LG31	405	184.71	0.46	6.07
Total	7627	5232.42	0.69	15.96

The quality of the genetic map was evaluated with heat maps and haplotype maps generated for each LG. The heat maps showed that there were no ordering errors detected in the 31 LGs of kelp. The heat map for LG6 is provided as additional Fig. S1. The haplotype maps confirmed that most of the recombination blocks in each LG were clearly defined. The haplotype map for LG6 is provided as additional Fig. S2.

### QTL analyses for blade length and blade width

**Figure 2:**
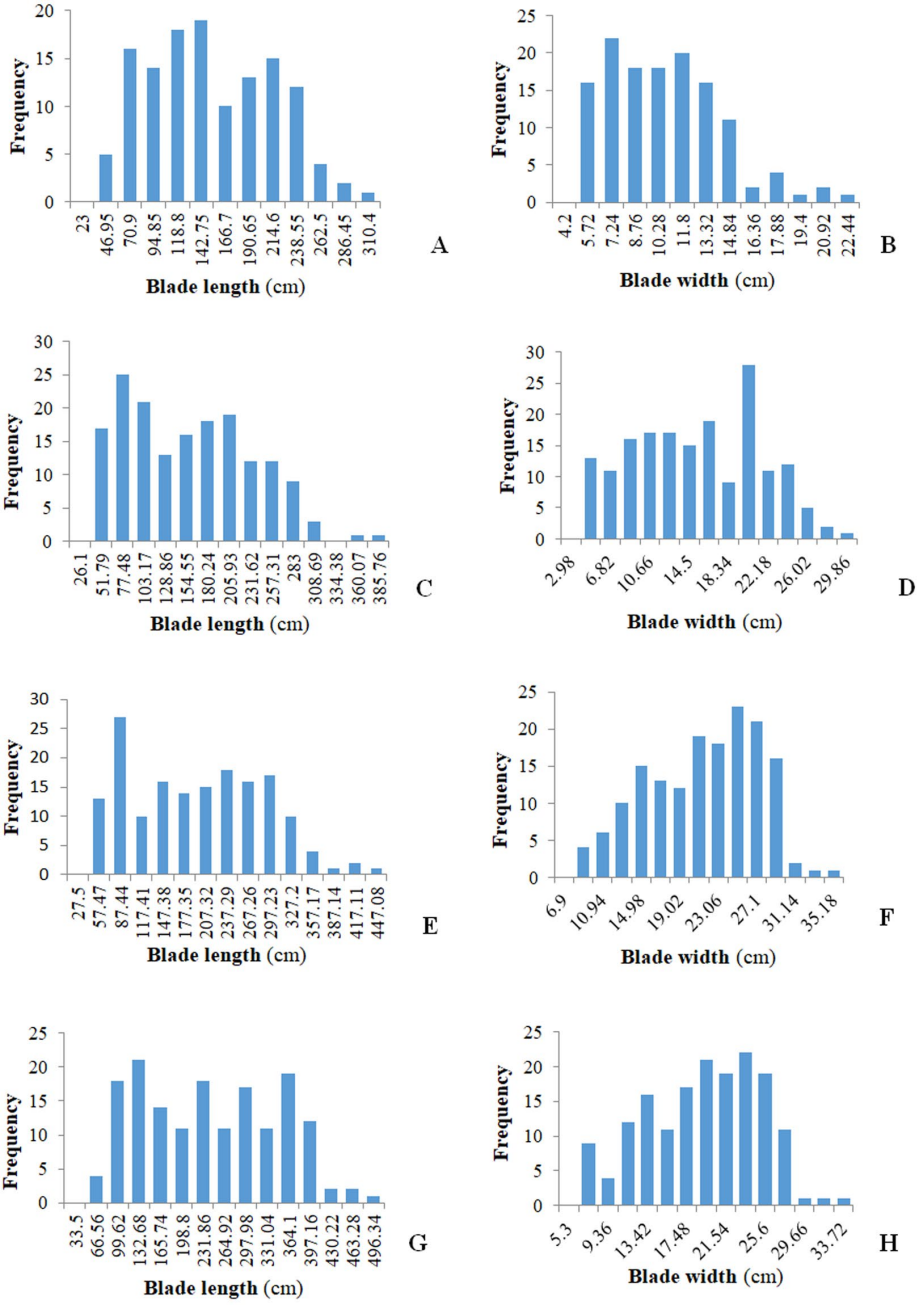
Frequency distribution patterns of the blade length and the blade width of *S*. *japonica* × *S. longissima* BC_1_F_2_ population at four serial observation days. (**A**) The blade length frequency distribution of *S*. *japonica* × *S. longissima* BC_1_F_2_ population measured at April 17^th^. (**B**) The blade width frequency distribution of *S.japoncica* × *S. longissima* BC_1_F_2_ population measured at April 17^th^. (**C**) The blade length frequency distribution of *S*. *japonica* × *S. longissima* BC_1_F2 population measured at May 8^th^. (**D**) The blade width frequency distribution of *S*. *japonica* × *S. longissima* BC_1_F_2_ populaltion measured at May 8^th^. (**E**) The blade length frequency distribution of *S*. *japonica* × *S. longissima* BC_1_F_2_ populaltion measured at May 22^th^. (**F**) The blade width frequency distribution of *S*. *japonica* × *S. longissima* BC_1_F_2_ populaltion measured at May 22^th^. (**G**) The blade length frequency distribution of *S*. *japonica* × *S. longissima* BC_1_F_2_ populaltion measured at June 9^th^. (**H**) The blade width frequency distribution of *S*. *japonica* × *S. longissima* BC^1^F^2^ populaltion meausred at June 9^th^.

Based on the high-density SNP linkage map and the phenotype data (Fig. 2; Table 2), we conducted QTL analyses of the blade length and width in kelp on the four days observed: April 17th, May 8th, May 22th and June 9th (Table 3). QTLs detected with LOD values above 2.5 using 1000 permutations were recognized as significant intervals. In total, 12 QTLs were detected for blade length and 10 for blade width (Table 3). Nevertheless, the total number of QTLs was only 18, indicating that some QTLs are likely pleiotropic. For example, the intervals between marker57879 and marker57878, and from LG7 was simultaneously detected for blade length and blade width on April 17. The QTL interval between marker52375 and marker52366 (LG15) was detected for blade width on May 22th and June 9th. In addition, the QTL interval between marker26129 and marker26127 (LG30) was detected for at least one trait on three observation dates. To assess the additive effects of each QTL in the kelp, the parental origins of the alleles at each QTL were evaluated. For qL4-5, the additive allele for increasing blade length originated in the female parent, *S*. *longissima*, while the additive allele of qW5-7 for increasing blade width originated in the male parent, “860”. Similar relationships were also found for qL10-4 and qW7-15. Interestingly, the allele from the female parent, *S*. *longissima*, could increase blade width at qW12-30 detected for width on June 9th, despite the fact that *S*. *longissima* has a narrow blade. Additionally, the allele from the male parent, which has a shorter blade, could increase blade length at qL6-24, which was mapped for blade length on April 17th. In addition, some QTLs were detected with obvious dominant values, which indicated that significant dominance existed between the alleles at these QTLs. The phenotypic variance explained by each designated QTL ranged from 5.37% to 18.69%. The most influential QTL interval was between marker26422 and marker53425 in LG24, accounting for 18.69% of the observed phenotypic variance with a LOD value of 5.53 (Table 3). The least effective QTL interval was between marker8613 and marker37947 in LG20 on May 22th, accounting for only 5.37% of the observed phenotypic variance with a LOD value of 2.52. The confidence intervals spanned by each mapped QTL ranged from 0.28 cM to 4.68 cM, with an average of 1.21 cM. The smallest interval was between marker26129 and marker26127 in LG30, and the largest interval was between marker5803 and marker5890 in LG2. The QTLs underlying the blade length and the blade width were also examined with the software QTL.gCIMapping from the R website, which adopted different genetic model and different statistical method for the QTL analysis from that in software QTL IciMapping, and the results were listed in Table S2. Although many QTLs detected by QTL.gCIMapping were different from that in Table 3, some QTLs still remained the same, such as the QTLs 1, 2, 3 for the blade length at April 17^th^ in Table S2.

**Table 2:** Summary statistics of the blade length and width in BC_1_F_2_ family on four observation days.

Date	Trait (cm)	Min	Max	Mean	SD	CV(%)	Skewness	Kurtosis
April 17th	FL	24.50	288.00	142.21	60.83	42.77	0.28	−0.81
	FW	4.30	21.00	9.96	3.58	35.94	0.69	0.24
May 8th	FL	26.20	360.20	144.64	75.43	52.15	0.35	−0.72
	FW	3.00	28.00	14.21	6.32	44.48	0.00	−1.00
May 22th	FL	27.60	417.00	186.43	92.30	49.51	0.11	−0.92
	FW	5.40	31.80	18.13	5.87	32.38	−0.29	−0.69
June 9th	FL	34.00	463.00	227.83	105.57	46.34	0.07	−1.07
	FW	7.00	33.20	20.65	6.00	29.06	−0.39	−0.76

**Table 3:** Details of QTLs detected for blade length (FL) and blade width (FW) in *S*. *japonica* on four observation days.

Date	Trait	QTL	LG	Position (cM)	Marker interval	Internal distance (cM)	LOD	Additive*	Dominant	PVE %
April 17th	FL	qL4-5	5	45.0	Marker69874-Marker60940	1.46	3.26	−0.04	39.78	11.52
		qL5-7	7	149.0	Marker57879-Marker57878	0.56	2.82	9.51	31.39	8.34
		qL6-24	24	154.0	Marker26422-Marker53425	1.74	5.53	14.11	−45.38	18.69
	FW	qW3-7	7	78.0	Marker44864-Marker13620	3.15	2.73	0.98	1.71	6.93
		qW4-7	7	144.0	Marker57906-Marker29834	1.37	3.10	1.14	1.58	8.16
		qW5-7	7	149.0	Marker57879-Marker57878	0.56	3.36	0.90	1.98	8.44
May 8th	FL	qL7–19	19	12.0	Marker18815-Marker18816	0.34	3.04	7.41	−42.74	9.07
		qL8-24	24	125.0	Marker73598-Marker48776	0.84	2.71	6.61	−37.82	7.59
	FW	qW6-30	30	79.0	Marker26129-Marker26127	0.28	2.96	−2.00	2.16	8.26
May 22th	FL	qL9-2	2	153.0	Marker5803-Marker5890	4.68	3.00	5.24	−47.82	6.57
		qL10-4	4	14.0	Marker56421-Marker56246	0.96	5.41	−47.57	−10.22	12.06
		qL11-20	20	0.0	Marker8613-Marker37947	1.80	2.52	−31.47	2.03	5.37
		qL12-22	22	0.0	Marker70109-Marker70108	0.90	3.66	−5.63	54.05	7.94
	FW	qW7-15	15	163.0	Marker52375-Marker52366	2.08	4.25	2.93	0.37	9.77
		qW8-30	30	79.0	Marker26129-Marker26127	0.28	3.34	−2.10	1.84	6.76
		qW9-30	30	97.0	Marker74604-Marker40179	0.28	2.90	−1.73	1.98	5.92
June 9th	FL	qL13-27	27	120.0	Marker19479-Marker19478	0.39	2.63	−31.32	36.31	5.46
		qL14-30	30	79.0	Marker26129-Marker26127	0.28	3.39	−39.25	37.73	6.87
		qL15-30	30	95.0	Marker71847-Marker56563	1.74	2.81	−29.31	45.18	5.94
	FW	qW10-1	1	58.0	Marker66438-Marker66442	0.28	2.51	0.54	2.92	6.20
		qW11-15	15	163.0	Marker52375-Marker52366	2.08	4.16	2.91	0.11	11.63
		qW12-30	30	77.0	Marker63372-Marker26139	0.56	3.87	−2.51	1.49	10.32

*A positive additive effect indicates that the male parent “860” allele increased the phenotypic value, and the female parent *S. longissima* allele decreased the phenotypic value, whereas a negative value indicates that the allele from “860” male parent decreased the phenotypic value, and the other allele from female parent *S. longissima* increased the phenotypic value.

### Candidate genes associated with blade length and blade width

**Figure 3:**
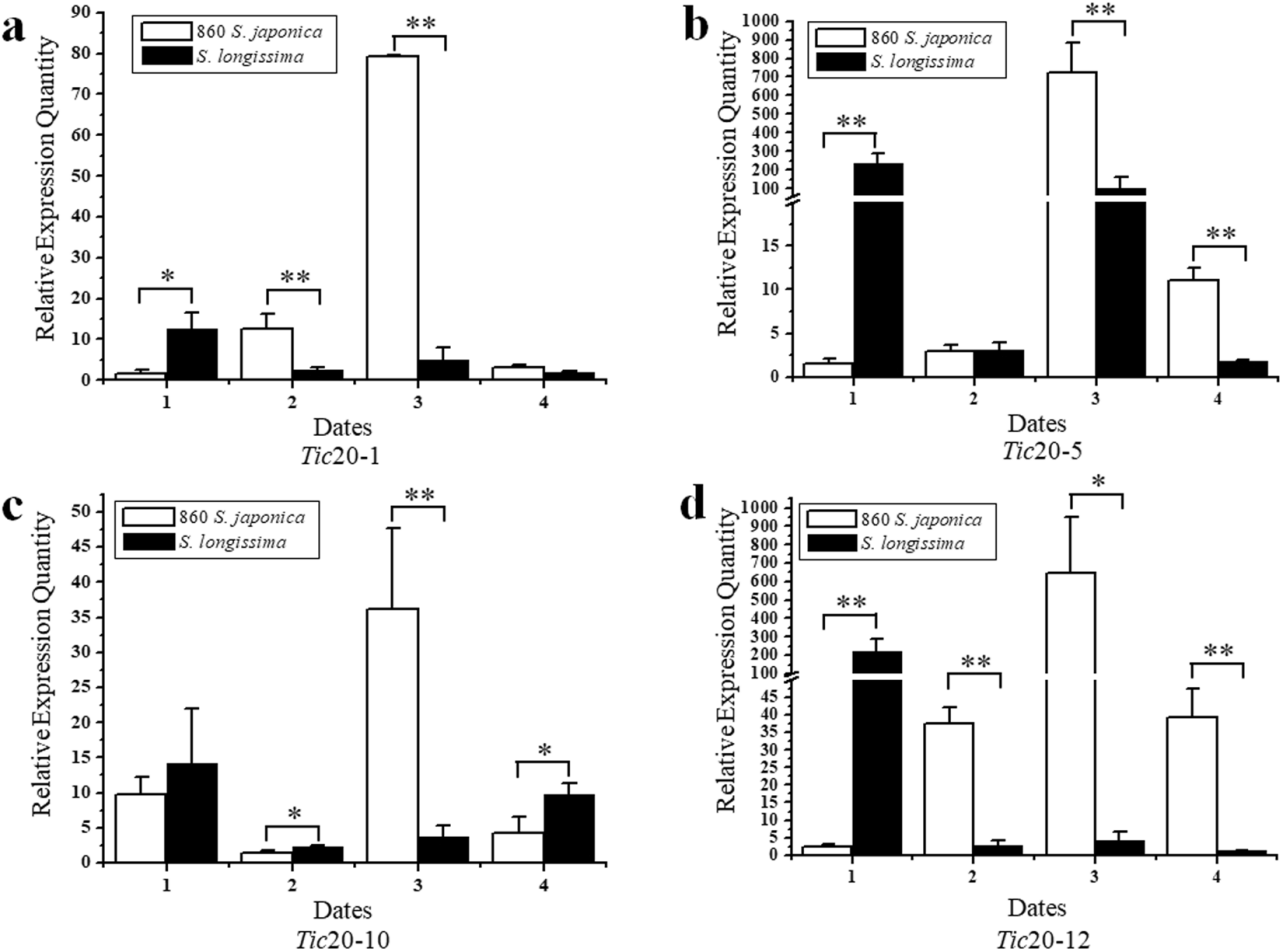
The expression pattern of four *Tic*20 genes in parent “860” and *S*. *longissima* on four observation days 1(April 16th), 2(May 6th), 3(July 2th), 4(July 24th). (**a**) *Tic20*-1 (**b**) *Tic20*-5 (**c**) *Tic20*-10 (**d**) *Tic20*-12. * and ** indicate significance level of P < 0.05 and 0.01, respectively.

Through comparison mapping, candidate genes were identified in the QTL intervals between marker26422 and marker53425 in LG24 and between marker26129 and marker26127 in LG30. Fourteen *Tic*20 genes were annotated between marker26422 and marker53425 in approximately 190 kb (208307~398831 in scaffold 192), and three peptidase genes were annotated between marker26129 and marker26127 in approximately 53 kb (210011~263935 in scaffold 182) (Table S3). The collinear relationship between LG30 and the reference genome including scaffold 182 was evaluated (Fig. S3). The proteins encoded by the *Tic*20 genes composed the TOC and TIC (translocon at the outer/inner envelope membrane of chloroplasts, respectively) complexes, which are responsible for translocating precursor proteins synthesized in the cytosol across or into the double chloroplast membrane envelope^41^. The enzymes encoded by the three genes of peptidases s8 and s53 belong to clan SB serine peptidases^42^. Transcriptional analysis of four selected *Tic*20 genes in the parental sporophytes was performed with RT-qPCR on the four growth dates; the results showed that the expression of these genes was different not only between the parent sporophytes but also between the four growth dates (Fig. 3). At the third observation day, for male parent “860” of *S*. *japonica*, each of the four *Tic*20 genes had the highest expression level and the expression level of *Tic*20-1 was significantly higher than that of female parent *S*. *longissima* (16.3 fold, *P* < 0.01). For *Tic*20-1, the expression level at the third observation day was significantly higher than that of the second observation day in “860” of *S*. *japonica* (7.9 fold, *P* < 0.01).

## Discussions

The collinearity of a high-density genetic linkage map with a reference genome can be used to enable the fine mapping of quantitative traits. In soybean, one map consisting of 5785 SNPs was constructed to identify the genes encoding isoflavone biosynthetic enzymes, with the detected QTL then comparatively mapped to the soybean reference genome^43^. Similarly, a high-density genetic map with 3441 SNP markers was generated for apple, and 80, 64, 17 genes related to fruit weight, fruit firmness and fruit acidity, respectively, were detected, after the relevant QTLs were comparatively mapped to the complete apple genome^44^. A comparative mapping strategy based on next-generation sequencing has also been attempted for localization of one “mut” locus for impaired branching pattern in *Ectocarpus siliculosus*^45^. Here, in *S*. *japonica*, we constructed a high-density genetic linkage map based on 7627 SNPs with an average genetic distance of 0.69 cM. We used this map to perform fine mapping for QTLs related to blade length and blade width in *S*. *japonica*. Eventually, 14 *Tic*20 genes and three peptidase s8 and s53 genes were identified as being associated with blade length and width in *S*. *japonica*. Thus, the colinearity of high-density genetic linkage maps with reference genomes can be a feasible and efficient way to identify candidate genes for quantitative traits.

A genetic correlation was detected between blade length and blade width in *S*. *japonica* in this study. The correlation coefficient between these two traits ranged from 0.80 to 0.92 (Table S4). Similarly, Wang (1984) reported that the phenotype correlation coefficient between blade length and blade width in *S*. *japonica* ranged from 0.81 to 0.95^16^. These results provide further evidence that phenotypically correlated traits are often mapped together, such as frond length and width in *Pyropia haitanensis*^26^ and kernel number per spike and thousand-kernel weight in wheat^46^. Liu *et al*.^20^ believed that the QTLs determining blade length and width in *S*. *japonica* were linked and located in the same chromosome region, but these authors did not detect a close correlation between blade length and blade width QTLs in their study^20^. In contrast, our study found that many QTLs determining both blade length and width were mapped to the same areas, such as the interval between marker57879 and marker57878 (Table 3). This indicated that the close phenotypic correlation of blade length and width in *S*. *japonica* was due mostly to genetic correlation, although environmental influences may also play a role. Due to the positive genetic correlation of blade length and blade width in *S*. *japonica*, selection for blade length could cause concurrent changes in blade width. Therefore, the identified locations of QTLs controlling both blade length and blade width in *S*. *japonica* should provide markers for more efficient marker-assisted selection (MAS) for these two traits in future breeding.

The genetically derived phenotypic correlation between blade length and width may be attributed to pleiotropy or to linkage disequilibrium^47^. Even though some QTLs for blade length and blade width were co-localized in the same genomic regions in this study, the possibility that the genes in the mapped QTLs are pleiotropic for these two traits must be further verified. The effects observed thus far might instead be due to linkage disequilibrium between different genes determining these two traits.

Fourteen *Tic*20 genes and three peptidase s8 and s53 genes were annotated to the QTL intervals between marker26422 and marker53425 in LG24 and between marker26129 and marker26127 in LG30, respectively. However, this finding does not exclude that the existence of other genes or regulators associated with blade length and blade width in *S*. *japonica* because the data used in this study were incomplete; the kelp genome contains some gaps in the contigs, and the correct assembly of some scaffolds remains uncertain.

The genes identified from the peptidase s8 and s53 gene families may be candidate genes for selection and breeding in kelp. In rice, one GS5 QTL was found to encode a putative serine carboxypeptidase belonging to the peptidase S10 family that contained a promoter region reported to be associated with grain width^48^. The peptidase s8 and s53 gene families are believed to have undergone a large evolutionary expansion in brown algae, and population genomic studies have showed that these gene families were artificially selected during the domestication of *S*. *japonica*^49^. Further research should explore the direct relation of these genes to blade length and width.

The expression differences in four *Tic*20 genes in the parental genotypes on the four cultivation dates were examined (Fig. 3). Whether these *Tic*20 genes are involved in determining blade length and blade width in *S*. *japonica*, remains to be verified. In the future, NIL (near isogenic lines) or CSSL (chromosome segment substitute lines) could be constructed to allow map-based cloning of the genes in the QTL region, or RNAi could be employed to verify the functions of these genes in kelp growth^50,51^.

In conclusion, a high-density genetic linkage map with 7627 SNPs was constructed for QTL analyses of blade length and width in *S*. *japonica*. A lot of 12 QTLs were found to be associated with blade length and 10 with blade width. The candidate genes mapped to these QTL intervals included 14 *Tic*20 genes and three peptidase s8 and s53 genes, which were proposed as candidate genes for blade length and width in *S*. *japonica*. The identified locations of QTLs controlling both blade length and width in *S*. *japonica* should lead to markers allowing for more efficient MAS for these two traits in future breeding.

## Electronic supplementary material

supplementary information

supplementary Dataset

## Data Availability

The SLAF reads of 178 individual sporophytes of “860” *S*. *japonica* × *S*. *longissima* BC_1_F_2_ population and parental gametophytes have been registered in NCBI with a bioproject accession number PRJNA413869 (https://www.ncbi.nlm.nih.gov/bioproject/413869).

## References

[1] Tseng CK (2001). Algal biotechnology industries and research activities in China. J. Appl. Phycol..

[2] Lane CE (2006). A multi-gene molecular investigation of the kelp (Laminariales, Phaeophyceae) supports substantial taxonomic re-organization. J. Phycol..

[3] Zhang J (2015). Phylogeographic data revealed shallow genetic structure in the kelp *Saccharina japonica* (Laminariales, Phaeophyta). BMC Evolutionary Biology.

[4] Van den Hoek, C. *et al*. Algae: An Introduction to Phycology. Cambridge University Press, Cambridge (1995).

[5] McHugh, D. J. A guide to the seaweed industry. FAO, Rome (2003).

[6] Mourisen, O G. Seaweed: edible, available, and sustainable. The University of Chicago Press, Ltd., London (2013).

[7] Food and Agriculture Organization of the United Nations. *Fisheries and Aquaculture Information and Statistics Services*. (2014).

[8] Fang TC, Li JJ (1966). The breeding of a long-frond variety of *Laminaria japonica* Aresch. Oceanologia et Limnologia Sinica.

[9] Wu CY, Lin GH (1987). Progress in the genetics and breeding of economic seaweeds in China. Hydrobiologia.

[10] Zhang QS (2007). Breeding of an elite laminaria variety 90-1 through inter-specific gametophyte clone of *Laminaria longissima* (Laminariales, Phaeophyta) and a female one of *L*. *japonica*. J. Appl. Phycol..

[11] Liu FL (2014). Breeding, economic traits evaluation, and commercial cultivation of a new *Saccharina* variety “Huangguan No. 1”. Aquacult. Int..

[12] Li XJ (2016). Improving seedless kelp (*Saccharina japonica*) during its domestication by hybridizing gametophytes and seedling raising from sporophytes. Scientific Reports.

[13] Zhang J (2011). Study on high-temperature-resistant and high-yield *Laminaria* variety “Rongfu”. J. Appl. Phycol..

[14] Fang TC (1983). Genetic studies of *Laminaria japonica* Aresch in China. Acta Ocenologica Sinica.

[15] Fang TC (1965). Further studies of the genetics of *Laminaria* frond length. Oceanologia et Limnologia Sinica.

[16] Wang QY (1984). A study of the heritability and genotypic correlation of some economic characters of *Laminaria japonica* Aresch. Journal of Shandong College of Oceanology.

[17] Chen JX (1983). The genetic studies of several main quantitative characters in *Laminaria japonica* (Aresch). Marine Fisheries Research.

[18] Yang GP (2009). Construction and characterization of a tentative amplified fragment length polymorphism-simple sequence repeat linkage map of *Laminaria* (Laminarials, Phaeophyta). J.Phycol..

[19] Liu FL (2009). Genetic mapping of the *Laminaria japonica* (Laminariales, Phaeophyta) using amplified fragments length polymorphism markers. J. Phycol..

[20] Liu FL (2010). QTL mapping for frond length and width in *Laminaria japonica* Aresch (Laminarales, Phaeophyta) using AFLP and SSR. Marine Biotechnol..

[21] Liu FL (2011). Identification of SCAR marker linking to longer frond length of *Saccharina japonica* (Laminariales, Phaeophyta) using bulked-segregant analysis. J. Appl. Phycol..

[22] Zhang J (2015). Genetic map construction and quantitative trait locus (QTL) detection of six economic traits using an F_2_ population of the hybrid from *Saccharina longissima* and *Saccharina japonica*. PLoS ONE.

[23] Ganal MW (2009). SNP identification in crop plants. Current Opinion in Plant Biology.

[24] Xu X, Bai G (2015). Whole-genome resequencing: changing the paradigms of SNP detection, molecular mapping and gene discovery. Mol. Breeding.

[25] Sun X (2013). SLAF-seq: an efficient method of large scale de novo SNP discovery and genotyping using high-throughput sequencing. PLoS ONE.

[26] Xu Y (2015). *et al*. Construction of a dense genetic linkage map and mapping quantitative trait loci for economic traits of a doubled haploid population of *Pyropia haitanensis* (Bangiales, Rhodophyta). BMC Plant Biology.

[27] Shan TF (2015). Construction of a high-density genetic map and mapping of a sex-linked locus for the brown alga *Undaria pinnatifida* (Phaeophyceae) based on large scale marker development by specific length amplified fragment (SLAF) sequencing. BMC Genomics.

[28] Zhang N (2015). Construction of a high density SNP linkage map of kelp (*Saccharina japonica*) by sequencing *Taq* I site associated DNA and mapping of a sex determining locus. BMC Genomics.

[29] Wang XL (2004). DNA fingerprinting of selected *Laminaria* (Phaeophyta) gametophytes by RAPD markers. Aquaculture.

[30] Li XJ (2007). Trait evaluation and trait cultivation of Dongfang no. 2, the hybrid of a male gametophyte clone of *Laminaria longissima* (Laminariales, Phaeophyta) and a female one of *L*. *japonica*. J. Appl. Phycol..

[31] Cock JM (2010). The *Ectocarpus* genome and the independent evolution of multicellularity in brown algae. Nature.

[32] Li H, Durbin R (2009). Fast and accurate short read alignment with Burrows-Wheeler transform. Bioinformatics.

[33] Li H (2009). The sequence alignment/map format and SAMtools. Bioinformatics.

[34] Xie S (2014). Linkage group correction using epistatic distorted markers in F_2_ and backcross populations. Heredity.

[35] Liu DY (2014). Construction and analysis of high-density linkage map using high-throughput sequencing. PLoS ONE.

[36] West MA (2006). High-density haplotyping with microarray-based expression and single feature polymorphism markers in *Arabidopsis*. Genome Res..

[37] Li H (2007). A modified algorithm for the improvement of composite interval mapping. Genetics.

[38] Wen, Y.-J. *et al*. An efficient multi-locus mixed model framework for the detection of small and linked QTLs in F2. *Briefings in Bioinformatics*, bby058 (2018) 10.1093/bib/bby058 PMC6917223 30032279

[39] Yao JT (2009). Improved RNA isolation for *Laminaria japonica* Aresch (Laminariaceae, Phaeophyta). J. Appl. Phycol..

[40] Shao ZR (2014). Characterization of mannitol-2-dehydrogenase in *Saccharina japonica*: evidence for a new polyol-specific long-chain dehydrogenases/reductase. PLoS ONE.

[41] Li H, Chiu C (2010). Protein transport into chloroplasts. Annu. Rev. Plant Biol..

[42] Rawlings, N. D. & Barrett, A. J. Handbook of Proteolytic Enzymes. Elsevier, London (2004).

[43] Li BL (2014). Construction of a high-density genetic map based on large-scale markers developed by specific length amplified fragment sequencing (SLAF-seq) and its application to QTL analysis for isoflavone content in *Glycine* max. BMC Genom..

[44] Sun R (2015). A dense SNP genetic map constructed using restriction site-associated DNA sequencing enables detection of QTLs controlling apple fruit quality. BMC Genomics.

[45] Billoud B (2014). Localization of causal locus in the genome of the brown macroalga *Ectocarpus*: NGS-based mapping and positioinal cloning approaches. Front. Plant Sci..

[46] Li C (2015). Single nucleotide polymorphism markers linked to QTL for wheat yield traits. Euphytica.

[47] Hallauer, A.R. *et al*. Quantitative genetics in maize breeding. Springer, New York (2010).

[48] Li Y (2011). Natural variation in *GS5* plays an important role in regulating grain size and yield in rice. Nat. Genet..

[49] Ye NH (2015). *Saccharina* genomes provide novel insight into kelp biology. Nature Communication.

[50] Takahashi F (2007). AUREOCHROME, a photoreceptor required for photomorphogenesis in stramenopiles. Proc. Natl. Acad. Sci. USA.

[51] Farnham G (2013). Gene silence in *Fucus* embryos: developmental consequences of RNAi-mediated cytoskeletal disruption. J. Phycol..

