# Supplementary material for: High-density SNP-based QTL mapping and candidate gene screening for yield-related blade length and width in Saccharina japonica (Laminariales, Phaeophyta)
Source: Sci Rep. 2018 Sep 11;8:13591. doi: 10.1038/s41598-018-32015-y (PMC6133921; doi:10.1038/s41598-018-32015-y)
Supplement: Supplementary file 1 — supplementary information [file 41598_2018_32015_MOESM1_ESM.doc]

High-density SNP-based QTL mapping and candidate gene screening for yield-related blade length and width in *Saccharina japonica* (Laminariales, Phaeophyta)

Xiuliang Wang 1,2,4[[1]](#footnote-2)*, Zhihang Chen ^1,2,3*^, Qiuying Li ^1,2,3^, Jie Zhang ^1,2,4^, Shun Liu^1,2,3^, Delin Duan ^1,2,4^

^1^Key Lab of Experimental Marine Biology, Institute of Oceanology, Chinese Academy of Sciences, Qingdao 266071, China

^2^Lab for Marine Biology and Biotechnology, Qingdao National Laboratory for Marine Science and Technology, Qingdao 266071, China

^3^University of Chinese Academy of Sciences, Beijing 100093, China

^4^Center of Ocean Mega-Science, Chinese Academy of Sciences, 7 Nanhai Road, Qingdao 266071, China

Correspondence and requests for materials should be addressed to D.L. Duan (dlduan@qdio.ac.cn).

Table S1. The primers used in RT-qPCR.

| Gene | Forward primer (5’→3’) | Reverse primer (5’→3’) |
| --- | --- | --- |
| *Tic20*-1 | GTGTGGTGTTCCGTGTTTG | GCTCTGGATACATCCGTACTT |
| *Tic20*-5 | TGGATCGAGATGGCAGTTT | ACGACGGGAACATGCAAT |
| *Tic20*-10 | CGGCAAGTGCTTCACATTTC | TATCAACCGATGCAACTACGG |
| *Tic20*-12 | GGCGGTTTCAGTTGTTTGTTC | CCGCTCGAAGCATTCCAATA |

Table S2. Details of QTLs detected for blade length (FL) and blade width (FW) in *S. japonica* detected by QTL.gCIMapping.

| Date | Trait | QTL | LG | Position (cM) | Marker interval | Internal distance (cM) | LOD | Additive | Dominant | PVE % |
| --- | --- | --- | --- | --- | --- | --- | --- | --- | --- | --- |
| April 17th | FL | 1 | 5 | 45.46 | Marker60946-Marker60946 | 0 | 3.77 | 0 | 22.90 | 3.54 |
|  |  | 2 | 7 | 149.33 | Marker57878-Marker57878 | 0 | 5.33 | 0 | 31.00 | 6.49 |
|  |  | 3 | 24 | 153.0 | Marker26422-Marker53425 | 1.74 | 7.92 | 0 | -38.39 | 9.96 |
| May 22th | FL | 1 | 2 | 153.0 | Marker5803-Marker5890 | 4.68 | 4.77 | 0 | -29.64 | 2.58 |
|  |  | 2 | 4 | 23.77 | Marker48364-Marker48364 | 0 | 4.45 | -30.34 | 0 | 5.40 |
|  |  | 3 | 7 | 149.33 | Marker57878-Marker57878 | 0 | 4.04 | 0 | 27.07 | 2.15 |
|  |  | 4 | 14 | 29.44 | Marker46732-Marker46732 | 0 | 3.93 | 0 | 25.59 | 1.92 |
|  |  | 5 | 22 | 0.0 | Marker70109-Marker70109 | 0 | 6.36 | 0 | 37.88 | 4.21 |
|  |  | 6 | 24 | 124.43 | Marker73598-Marker73598 | 0 | 5.37 | 0 | -32.98 | 3.19 |
|  |  | 7 | 27 | 8 | Marker17687-Marker17691 | 2.02 | 3.59 | 0 | 25.19 | 1.86 |
|  |  | 8 | 30 | 59.32 | Marker29584-Marker29584 | 0 | 2.56 | -19.57 | 0 | 2.25 |
|  |  | 9 | 31 | 158.29 | Marker57107-Marker57107 | 0 | 5.15 | 0 | 32.92 | 3.18 |
| June 9th | FW | 1 | 15 | 162.51 | Marker52375-Marker52375 | 0 | 4.09 | 2.25 | 0 | 7.02 |
|  |  | 2 | 30 | 78.71 | Marker26126-Marker26126 | 0 | 3.13 | -1.50 | 0 | 3.14 |

Table S3. Candidate genes associated with the blade length and width in *S. japonica*.

| QTL interval | Genes | Register number in NCBI |
| --- | --- | --- |
| marker26422 ~ marker53425 | *Tic20*-1 | KY411547 |
|  | *Tic20*-2 | KY411548 |
|  | *Tic20*-3 | KY411549 |
|  | *Tic20*-4 | KY411550 |
|  | *Tic20*-5 | KY411551 |
|  | *Tic20*-6 | KY411552 |
|  | *Tic20*-7 | KY411553 |
|  | *Tic20*-8 | KY411554 |
|  | *Tic20*-9 | KY411555 |
|  | *Tic20*-10 | KY411556 |
|  | *Tic20*-11 | KY411557 |
|  | *Tic20*-12 | KY411558 |
|  | *Tic20*-13 | KY411559 |
|  | *Tic20*-14 | KY411560 |
| marker26129 ~ marker26127 | Peptidase S8 and S53-1 | KY411544 |
|  | Peptidase S8 and S53-2 | KY411545 |
|  | Peptidase S8 and S53-3 | KY411546 |

Table S4. Correlation between the blade length and width in the BC_1_F_2_ population.

| Date | Correlation coefficient |
| --- | --- |
| April 17th | 0.84 |
| May 8th | 0.92 |
| May 22th | 0.89 |
| June 9th | 0.88 |


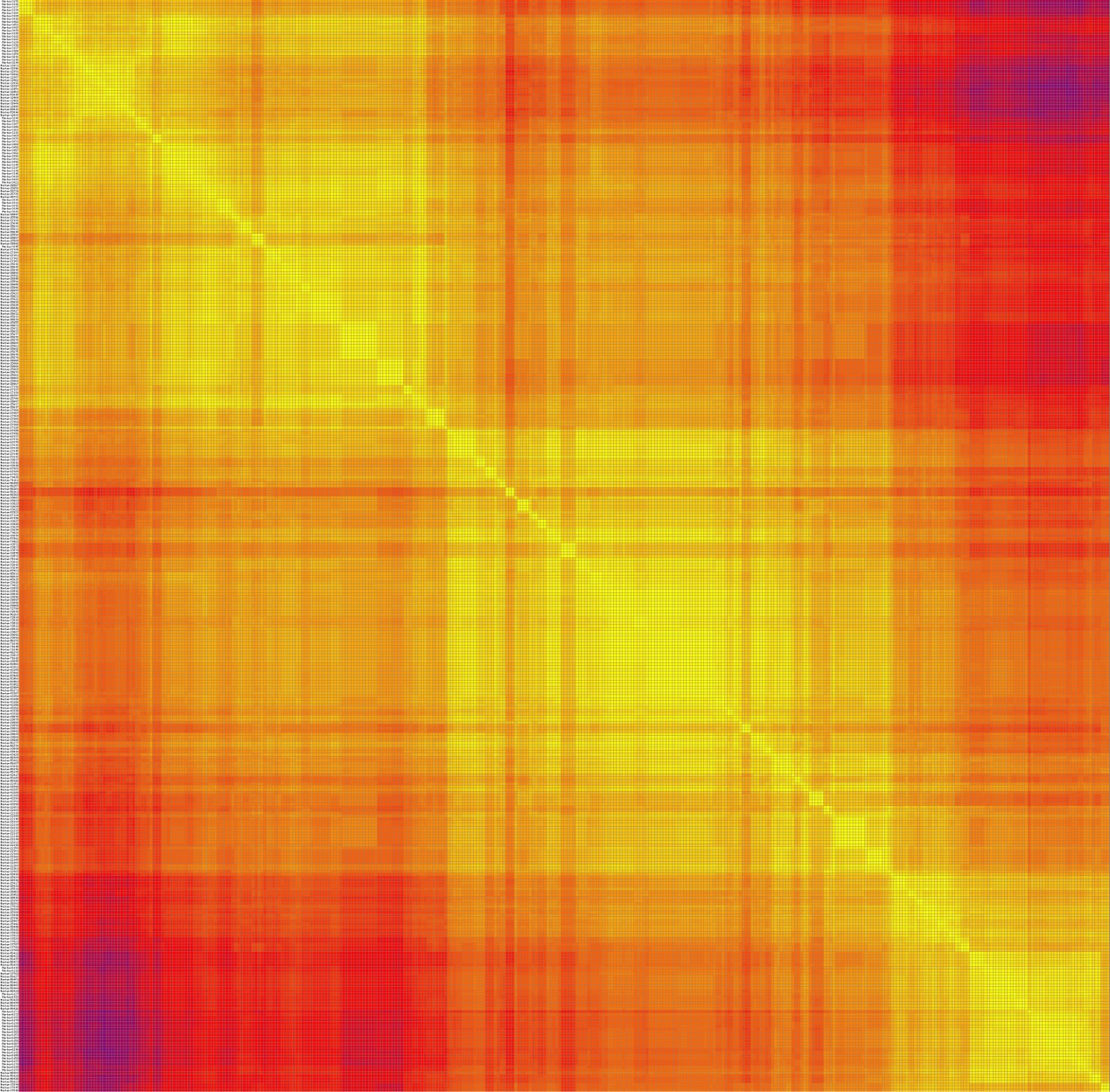
Figure S1. Heat map for LG6. Each cell represents the recombination rate between two markers. The color change from yellow through red to purple indicates the change of recombination rate from low to high.


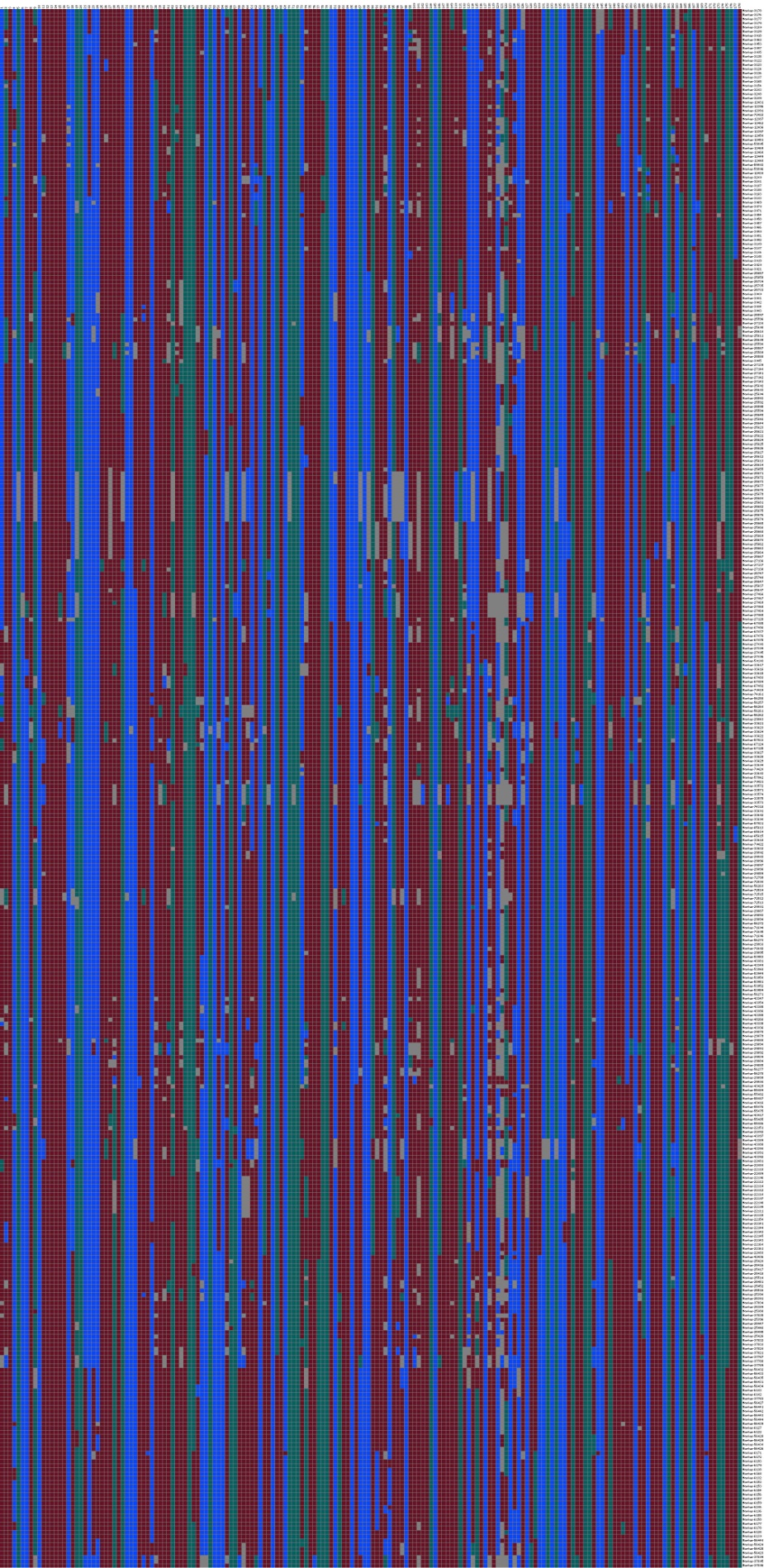


Figure S2. Haplotype map for LG6. Blue and red represent markers originating from maternal and paternal parent, respectively. Gray represents missing data. Rows indicate the markers on the linkage group and colums indicates the genotype of an individual.


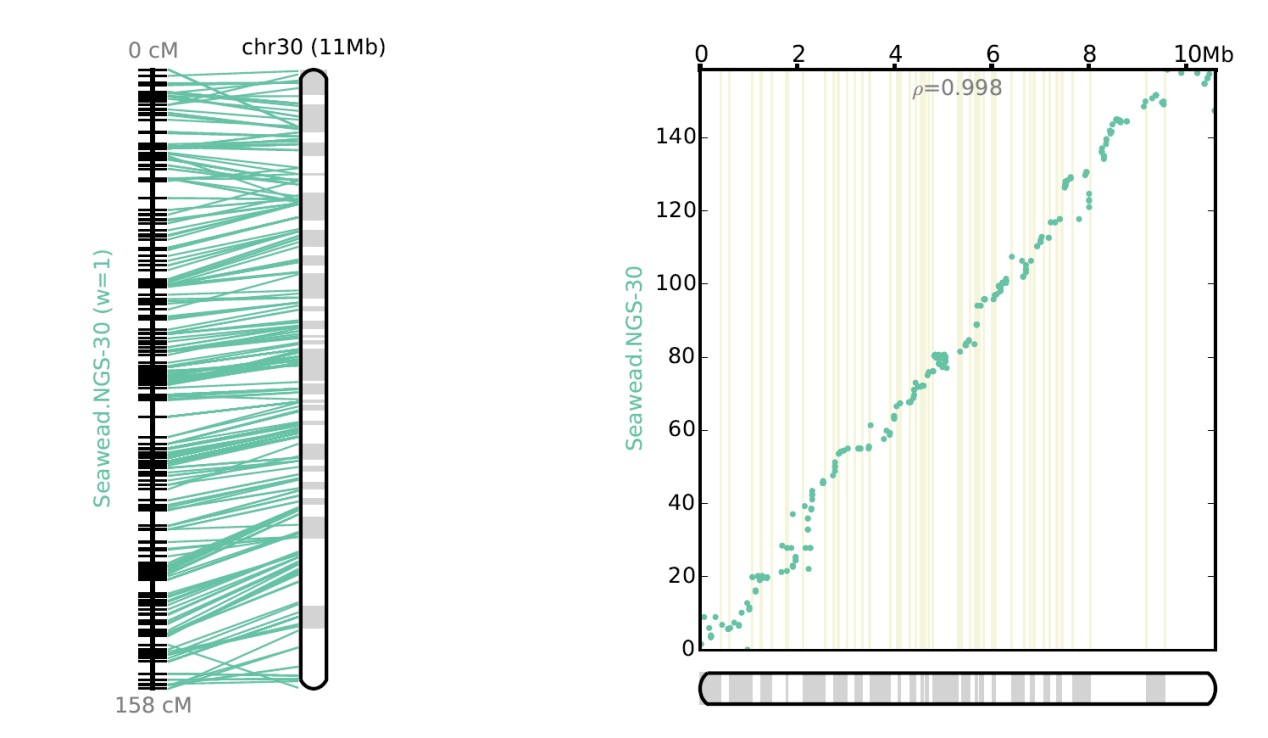


Figure S3. The collinearity plot of LG30 with the kelp reference genome.

1. ^^equal contribution. [↑](#footnote-ref-2)
